# Efficacy of Antipsychotic Augmentation Therapy in Treatment-Resistant Obsessive-Compulsive Disorder: A Systematic Review and Meta-Analysis

**DOI:** 10.7759/cureus.108358

**Published:** 2026-05-06

**Authors:** Ammar Shahtou, Hend R Omara, Sherin A Qari, Rand S Alqahtani, Ziyad S Alnasyan, Abdulkhaliq E Alotaibi, Ammar A Ali, Jehad M Alabdulrahim, Sara A Naser, Nada Asad, Lolwah Bagabas, Sara A Jilani, Ali S Metwaly

**Affiliations:** 1 Psychiatry, ERADAH Complex and Mental Health, Najran, SAU; 2 Neuropsychiatry, Menoufia University, Shebeen El-Kom, EGY; 3 Research and Development, King Abdullah International Medical Research Center, Jeddah, SAU; 4 College of Medicine, King Saud Bin Abdulaziz University for Health Sciences, Jeddah, SAU; 5 College of Medicine, Najran University, Najran, SAU; 6 College of Medicine, Qassim University, Qassim, SAU; 7 Psychiatry, King Abdulaziz University, Jeddah, SAU; 8 General Practice, Saudi Commission for Health Specialties, Mecca, SAU; 9 Psychiatry, Salmaniya Medical Complex, Manama, BHR; 10 College of Medicine, King Fahad Specialist Hospital, Dammam, SAU; 11 Psychiatry, King Saud Bin Abdulaziz University for Health Sciences, Jeddah, SAU; 12 Psychiatry, Fakeeh College for Medical Sciences, Jeddah, SAU; 13 Precision Medicine, Faculty of Pharmacy, Alexandria University, Alexandria, EGY

**Keywords:** antipsychotic augmentation, aripiprazole, network meta-analysis, obsessive-compulsive disorder, quetiapine, risperidone, systematic review, treatment-resistant

## Abstract

Obsessive-compulsive disorder (OCD) is a chronic neuropsychiatric condition. While selective serotonin reuptake inhibitors (SSRIs) are a standard treatment, many patients experience an inadequate response to monotherapy. For individuals with treatment-resistant OCD (TR-OCD), antipsychotic augmentation is a commonly utilized pharmacological strategy. This review aimed to systematically review the literature and conduct a frequentist network meta-analysis (NMA) to evaluate the comparative efficacy, safety, and tolerability of first-, second-, and third-generation antipsychotic augmentation in adults with TR-OCD. A systematic search was conducted from database inception to March 2026. Double-blind randomized controlled trials (RCTs) and observational studies evaluating antipsychotic augmentation of ongoing SRI therapy in adults with TR-OCD were included. The primary outcome was the mean change in the Yale-Brown Obsessive-Compulsive Scale (Y-BOCS) total score. The secondary outcomes included categorical treatment response (Y-BOCS reduction ≥ 35%) and tolerability. Pairwise meta-analyses utilized an inverse-variance random-effects model with Hartung-Knapp-Sidik-Jonkman (HKSJ) adjustment. The NMA established an empirical treatment hierarchy using surface under the cumulative ranking curve (SUCRA) probabilities. The certainty of the evidence was evaluated using the Grading of Recommendations Assessment, Development and Evaluation (GRADE) approach. Forty-three studies (22 RCTs and 21 observational studies) encompassing 890 patients were included. Pairwise meta-analysis of the RCTs demonstrated that, as a class, antipsychotic augmentation was significantly superior to placebo in reducing continuous Y-BOCS scores (standardized mean difference (SMD) = -0.49; 95% CI: -0.99 to -0.00; p < 0.05) and increasing the odds of categorical response (odds ratio (OR) = 2.38; 95% CI: 0.97 to 5.86). The NMA revealed a distinct hierarchy of efficacy: haloperidol exhibited the largest effect compared to placebo (SMD = -1.34; SUCRA = 0.799), followed by olanzapine (SMD = -0.80; SUCRA = 0.596), risperidone (SMD = -0.74; SUCRA = 0.625), and aripiprazole (SMD = -0.57; SUCRA = 0.532). The evidence for quetiapine (SMD = -0.45; SUCRA = 0.450) and paliperidone (SMD = -0.22; SUCRA = 0.356) was less robust and indistinguishable from that of the placebo. Although haloperidol was highly efficacious, its use was limited by poor tolerability. Risperidone and aripiprazole demonstrated the most optimal balance of robust anti-obsessional efficacy and acceptable tolerability. Preliminary data highlighted the potential of third-generation agents, such as brexpiprazole. Antipsychotic augmentation is a highly effective, evidence-based strategy for managing TR-OCD. Based on comparative efficacy and tolerability profiles, risperidone and aripiprazole are the preferred augmenting agents. The routine use of quetiapine should be reconsidered because of its marginal superiority over placebo. Future large-scale active-comparator RCTs are essential to define the long-term metabolic burden and evaluate novel agents to advance precision psychiatry in refractory OCD.

## Introduction and background

Obsessive-compulsive disorder (OCD) is a debilitating and chronic neuropsychiatric condition characterized by recurrent, intrusive, and distressing thoughts (obsessions) and repetitive ritualistic behaviours or mental acts (compulsions) [1]. Affecting approximately 1% to 3% of the global adult population, OCD emerges in early life and follows a waxing and waning course [2,3]. This disorder is one of the leading causes of non-fatal illness-related disability, exerting a toll on patients' psychosocial functioning, occupational attainment, and quality of life, while generating a socioeconomic burden [4,5]. Current international clinical guidelines establish selective serotonin reuptake inhibitors (SSRIs) and cognitive-behavioural therapy (CBT), specifically exposure and response prevention (ERP), as foundational first-line therapeutic interventions [6-8].

Despite the proven efficacy of these primary treatments, a substantial proportion of patients fail to achieve clinical remission. Epidemiological and clinical trial data indicate that 40% to 60% of patients with OCD exhibit an inadequate response to optimal trials of SSRI monotherapy [9-11]. Treatment-resistant OCD (TR-OCD), operationalized as the persistence of clinically significant symptoms (e.g., Yale-Brown Obsessive-Compulsive Scale (Y-BOCS) score ≥ 16) following at least two adequate trials of SSRIs or clomipramine, represents a formidable clinical challenge that leaves patients vulnerable to prolonged suffering and necessitates alternative pharmacological strategies [12-14].

Among the available next-step pharmacological approaches, the augmentation of ongoing serotonin reuptake inhibitor (SRI) therapy with antipsychotic medications is the most validated and widely prescribed intervention [15-17]. The mechanistic rationale for this approach centers on the modulation of the cortico-striato-thalamo-cortical (CSTC) circuitry [18,19]. The combined antagonism or partial agonism of dopamine D2 and serotonin 5-HT2A/5-HT1A receptors by these agents acts synergistically with SRIs to attenuate dopaminergic hyperactivation and potentiate anti-obsessional effects [20,21]. Conventional pairwise meta-analyses and early network meta-analyses (NMAs) have demonstrated the superior efficacy of specific antipsychotics, most notably risperidone, aripiprazole, and haloperidol, over placebo in reducing OCD symptom severity, with approximately one-third of refractory patients achieving a clinically meaningful response [22-25]. Atypical antipsychotic augmentation is recommended by major psychiatric associations as the primary evidence-based strategy for managing TR-OCD [6,26].

Critical gaps in the evidence persist, precluding the definitive determination of a singular optimal antipsychotic agent. Previous meta-analyses have yielded conflicting hierarchical rankings due to significant inter-study heterogeneity, small sample sizes, and a notable scarcity of head-to-head randomized controlled trials (RCTs) [24,27]. Furthermore, while therapeutic efficacy must be weighed against the metabolic and extrapyramidal burden of these agents [28], differences in tolerability and treatment discontinuation rates across antipsychotic classes remain inadequately synthesized. In addition, the clinical response to antipsychotic augmentation is highly heterogeneous and may be influenced by specific demographic and clinical moderators, such as baseline symptom severity and the presence of comorbid tic disorders [22,29]. Additionally, since the publication of the last major systematic reviews, several recent RCTs and observational studies have evaluated novel third-generation antipsychotics (e.g., brexpiprazole and cariprazine) and utilized optimized trial protocols [30]. However, the integration of these newer agents into the existing therapeutic hierarchy has not yet been comprehensively evaluated.

To address these critical evidence gaps, an updated systematic review and NMA of antipsychotic augmentation in adults with TR-OCD were conducted. The primary objective of this study was to synthesize and evaluate the comparative efficacy, safety, and tolerability of second- and third-generation antipsychotics. By establishing empirically derived treatment hierarchies, examining dose-response relationships, and investigating critical moderators of treatment response, such as comorbid tics, baseline severity, and prior pharmacological failures, this review aimed to generate contemporary recommendations to inform precision psychiatry and optimize clinical decision-making for this highly vulnerable patient population.

## Review

Methods

Protocol and Registration

This systematic review and meta-analysis were conducted in accordance with the Preferred Reporting Items for Systematic Reviews and Meta-Analyses (PRISMA) 2020 guidelines [31] and the PRISMA Extension for Network Meta-Analyses (PRISMA-NMA) [32]. The review protocol was prospectively registered in the International Prospective Register of Systematic Reviews (PROSPERO) under the identifier CRD420251207366.

Eligibility Criteria

The eligibility criteria were structured using the Population, Intervention, Comparator, Outcomes, and Study design (PICOS) framework. The population comprised adults (≥ 18 years of age) with a primary diagnosis of OCD according to the Diagnostic and Statistical Manual of Mental Disorders (DSM) or International Classification of Diseases (ICD) criteria. Patients had to meet the operational definition of TR-OCD, defined as an inadequate clinical response to at least two adequate trials of SSRIs or clomipramine at the maximum tolerated therapeutic dose for a minimum of 8 to 12 weeks. A baseline Y-BOCS score of ≥ 16 was required. The interventions were augmentation of ongoing SRI therapy with second-generation (e.g., aripiprazole, risperidone, olanzapine, quetiapine, and paliperidone) or third-generation (e.g., brexpiprazole and cariprazine) antipsychotic agents. First-generation antipsychotics (FGAs) (e.g., haloperidol) were included only if they were specifically investigated for SRI augmentation in TR-OCD. The comparators were placebo augmentation or standard SRI monotherapy. In the NMA, different antipsychotic agents served as active comparators.

The primary efficacy outcome was the magnitude of symptom reduction, measured as the mean change in the Y-BOCS total score from baseline to the endpoint. Secondary outcomes included categorical treatment response (operationally defined as a ≥ 35% reduction in Y-BOCS scores), remission rates, and treatment tolerability/safety (measured by all-cause discontinuation rates and specific adverse events). Double-blind RCTs and non-randomized, open-label prospective/retrospective cohort studies were included. Single case reports (n < 5) and studies restricted to the pediatric population were excluded.

Search Strategy and Selection Process

A systematic search of the literature was conducted across the following major bibliographic databases: CENTRAL (Cochrane Central Register of Controlled Trials), MEDLINE (via PubMed), Embase, PsycINFO, CINAHL, and Scopus, from database inception to the date of the search. The search strategy utilized a combination of Medical Subject Headings (MeSH) and free-text keywords, including combinations of "obsessive-compulsive disorder", "treatment-resistant", "antipsychotic augmentation", "aripiprazole", "risperidone", "olanzapine", "quetiapine", and "brexpiprazole". Trial registries (ClinicalTrials. gov, ISRCTN) were searched to capture gray literature and mitigate reporting and dissemination biases. Furthermore, backward and forward citation chaining of the included studies and relevant review articles was performed manually. Two independent reviewers screened the titles and abstracts, followed by a full-text evaluation. Discrepancies were resolved through consultation with a third reviewer.

Data Extraction and Risk of Bias Assessment

Data extraction was independently performed by two investigators using the standardized forms. The extracted variables included study characteristics, patient demographics, antipsychotic dosages, and baseline/endpoint outcome measures.

The risk of bias at the study level was evaluated independently by two reviewers. For RCTs, the Cochrane Risk of Bias 2.0 (RoB 2) tool was utilized to assess domains such as the randomization process, deviations from intended interventions, missing outcome data, measurement of the outcome, and selection of the reported result [33]. For non-randomized and observational studies, the Newcastle-Ottawa Scale (NOS) was employed to evaluate selection, comparability, and outcome ascertainment [34].

Statistical Analysis and Data Synthesis

All statistical analyses were performed using R software, version 4.5.2 (R Foundation for Statistical Computing, Vienna, Austria), utilizing the meta and metafor packages for pairwise meta-analyses [35] and the netmeta package for NMAs [36].

Effect Measures and Statistical Model

For continuous outcomes (e.g., Y-BOCS score reductions), the effect size index was calculated as the standardized mean difference (SMD) with 95% confidence intervals (CIs). For dichotomous outcomes (e.g., categorical responses and discontinuation rates), odds ratios (ORs) with 95% CIs were computed. To pool prevalence and proportion data effectively, we applied the Freeman-Tukey Double arcsine data transformation to stabilize variances before pooling [37]. Recognizing the clinical and methodological heterogeneity across the studies, an inverse-variance random-effects statistical model was employed. To ensure robustness and account for uncertainty in the variance estimates, especially given the anticipated small number of studies in certain comparisons, the Hartung-Knapp-Sidik-Jonkman (HKSJ) variance adjustment was applied [38].

NMA Framework

To establish empirically derived treatment hierarchies among the different antipsychotic classes, a frequentist NMA was conducted. Effect estimates were synthesized from both direct and indirect comparisons. The ranking of interventions was quantitatively determined using the surface under the cumulative ranking curve (SUCRA) probabilities [39].

Statistical Heterogeneity (Dispersion)

Statistical heterogeneity across studies was evaluated using Cochran’s Q test and quantified with the I^2^ statistic and τ^2^ (between-study variance) [40]. An I^2^ value > 50% was indicative of a substantial statistical inconsistency.

Moderators and Robustness (Sensitivity and Subgroup Analysis)

Prespecified subgroup analyses and meta-regressions (moderator analyses) were conducted to explore the sources of heterogeneity. The moderators investigated included antipsychotic generation (second vs. third generation), presence of comorbid tics, baseline OCD severity, and antipsychotic dosage. Sensitivity analyses were performed to test the robustness of the findings by restricting the pooled estimates exclusively to high-quality RCTs and sequentially employing a leave-one-out approach.

Assessment of Bias (Small-Study Effects)

The presence of small-study effects and potential publication bias was evaluated through a visual assessment of bias using funnel plots [41]. For objective confirmation, statistical tests for small study effects were conducted using Egger's linear regression test [42]. If asymmetry was detected, the Duval and Tweedie trim-and-fill procedure was used for adjustment.

Certainty/Strength of Evidence (Overall Body)

The overall certainty and strength of the evidence for the primary and secondary outcomes were evaluated using the Grading of Recommendations Assessment, Development, and Evaluation (GRADE) approach. Evidence was systematically downgraded based on limitations in study design (risk of bias), inconsistency (statistical heterogeneity), indirectness, imprecision, and publication bias, resulting in a final classification of high, moderate, low, or very low certainty [43].

Results

Study Selection and Characteristics

**Figure 1:**
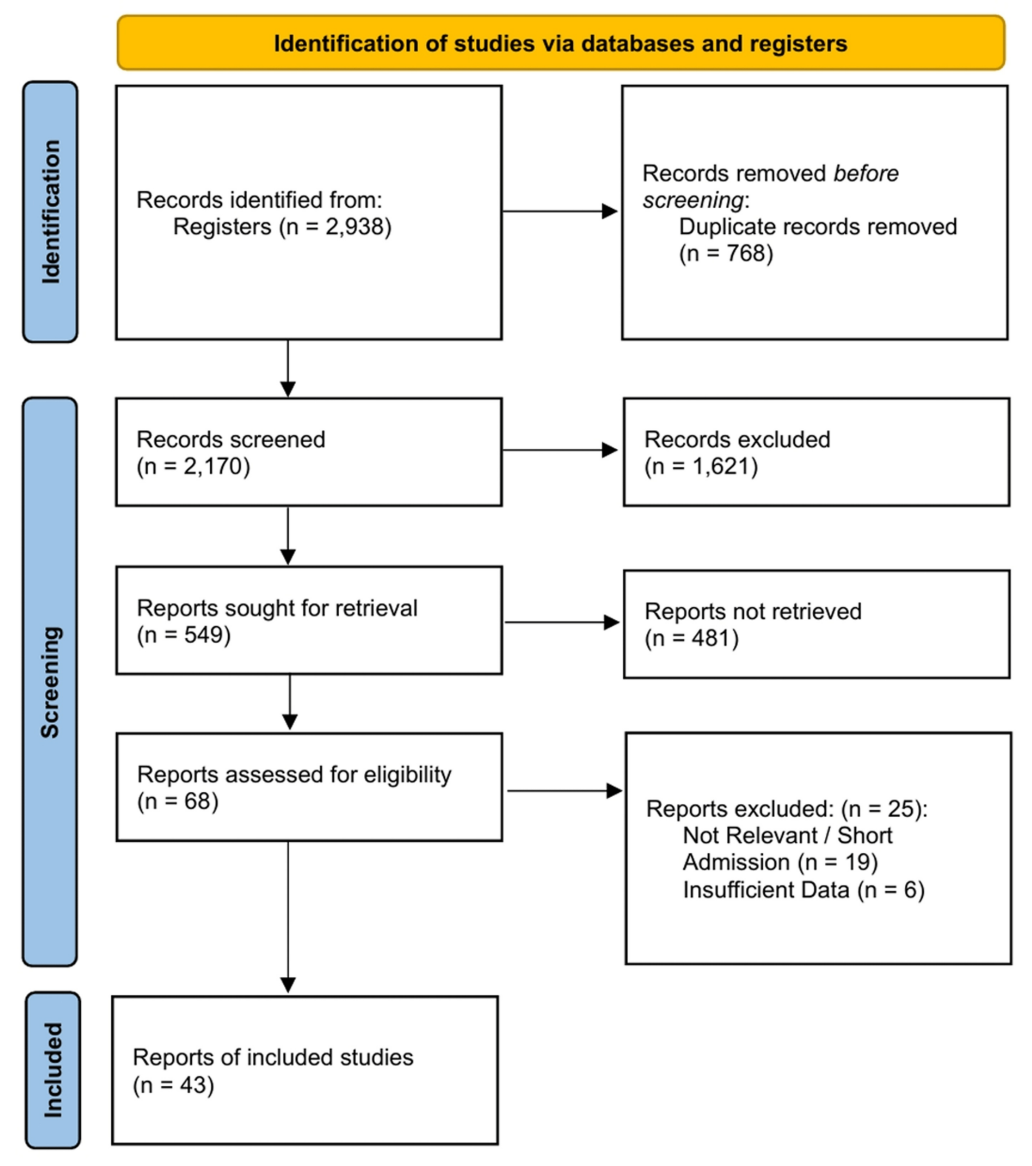
PRISMA 2020 flow diagram. PRISMA: Preferred Reporting Items for Systematic Reviews and Meta-Analyses

The systematic literature search across databases and registries initially yielded 2,938 records. After the removal of 768 duplicates, 2,170 records underwent title and abstract screenings. Of these, 1,621 were excluded from the study. We sought to retrieve the full texts of the remaining 549 reports, obtaining 68 for a detailed eligibility assessment. Following the full-text review, 25 reports were excluded (19 due to lack of relevance or short admission duration and six due to insufficient extractable data). Forty-three studies [13,24,29,44-83] were eligible for inclusion in the systematic review and meta-analysis. The study selection process is detailed in the PRISMA 2020 flowchart (Figure 1).

The characteristics of the included studies are presented in Table 1. This review encompasses both RCTs and non-randomized, open-label, or retrospective studies evaluating various antipsychotic augmentation strategies, including aripiprazole, haloperidol, olanzapine, paliperidone, quetiapine, and risperidone. The treatment duration ranged from four to 52 weeks, with baseline mean Y-BOCS scores indicating severe symptomatology (>24).

**Table 1:** Characteristics of included studies. RCT: randomized controlled trial; CBT: cognitive behavioral therapy; Pbo: placebo; Ris: risperidone; NR: not reported; Hal: haloperidol; Que: quetiapine; Ari: aripiprazole; Pal: paliperidone; Clomi: clomipramine; Ola: olanzapine; SD: standard deviation; Y-BOCS: Yale-Brown Obsessive Compulsive Scale; VAS: Visual Analogue Scale; OCD: obsessive-compulsive disorder; NR: not reported

Study ID	Study Design	Antipsychotic Agent(s)	Dose (mg/d)	N (Active/Control)	Duration (Weeks)	Baseline Y-BOCS (Mean ± SD)	Endpoint Y-BOCS (Mean ± SD)	Response Rate (%)
Simpson 2013 [13]	RCT	Risperidone/CBT/Pbo	1.9 ± 1.1	40/40/20	8	Ris: NR, Pbo: NR	Ris: NR, Pbo: NR	Ris: 22.5%, Pbo: 15%
McDougle 1994 [24]	RCT	Haloperidol/Pbo	6.2 ± 3.0	17/17	4	Hal: 25.4 ± 5.0, Pbo: 24.9 ± 4.0	Hal: 15.5 ± 9.1, Pbo: NR	Hal: 65%, Pbo: 0%
Carey 2012 [29]	Retrospective	Quetiapine	NR	NR	NR	NR	NR	NR
Atmaca 2002 [44]	RCT	Quetiapine/Pbo	50-200	14/13	8	Que: 24.1 ± 4.9, Pbo: 23.8 ± 4.1	Que: 13.4 ± 3.2, Pbo: 21.4 ± 4.3	Que: 71.4%, Pbo: 0%
Shafti 2019a [45]	Open-label	Olanzapine	2.5-10	11/0	8	33.45 ± 4.47	25.0 ± 5.98	54.5%
Shafti 2019b [46]	RCT	Aripiprazole/Quetiapine	Ari: 10, Que: 300	22/22	12	Ari: 33.17 ± 3.9, Que: 31.18 ± 4.9	Ari: 30.72 ± 4.6, Que: 27.97 ± 3.7	Ari: 27.3%, Que: 54.5%
Farhan 2012 [47]	Open-label	Olanzapine	2.5-5	20/0	12	33.4 ± 3.9	21.7 ± 3.3	80%
Koran 2000 [48]	Open-label	Olanzapine	2.5-10	10/0	8	29.0 ± 4.9	24.4 ± 8.0	30%
Weiss 1999 [49]	Case Series	Olanzapine	NR	NR	NR	NR	NR	NR
Delle Chiaie 2011 [50]	Open-label	Aripiprazole	12.6 ± 4.2	20/0	12	28.99 ± 3.84	15.55 ± 5.47	80%
Pfanner 2000 [51]	Open-label	Risperidone	3	20/0	8	36.1 ± 3.4	24.8 ± 3.6	NR
D'Amico 2003 [52]	Open-label	Olanzapine	5-10	18/0	12	27.1 ± 4.0	20.1 ± 3.9	38.9%
Assarian 2018 [53]	RCT	Aripiprazole/Risperidone	Ari: 10, Ris: 2	44/43	12	Ari: 25.0 ± 4.4, Ris: 25.2 ± 4.1	Ari: 16.2 ± 4.4, Ris: 20.0 ± 4.4	NR
Hegde 2017 [54]	Retrospective	Aripiprazole	9.5 ± 3.3	23/0	NR	21.43 ± 7.64	NR	26%
Storch 2013 [55]	RCT	Paliperidone/Pbo	4.9 ± 2.3	17/17	8	Pal: 27.1 ± 5.6, Pbo: 25.1 ± 4.3	Pal: 19.1 ± 11.1, Pbo: 21.2 ± 8.1	Pal: 35%, Pbo: 18%
Denys 2004 [56]	RCT	Quetiapine/Pbo	≤ 300	20/21	8	Que: 26.4 ± 4.6, Pbo: 27.7 ± 3.9	Que: 19.3 ± 7.2, Pbo: 20.5 ± 8.4	Que: 40%, Pbo: 47.6%
Diniz 2011 [57]	RCT	Quetiapine/Clomi/Pbo	Que: 142 ± 65	18/18/18	12	Que: 25 ± 6 (Final)	Que: 25 ± 6, Pbo: 18 ± 7	Que: 33%, Pbo: 56%
Denys 2002 [58]	Open-label	Quetiapine	NR	10/0	NR	31.4 ± 7.8	20.8 ± 8.4	NR
Giacovelli 2025 [59]	Retrospective	Brexpiprazole	1-2	10/0	12	28.1 ± 2.4	18.3 ± 6.0	70%
Bogetto 2000 [60]	Open-label	Olanzapine	5	23/0	12	26.8 ± 3.0	18.9 ± 5.9	43.5%
Stein 2009 [61]	Open-label	Atypical (mixed)	NR	46/0	52	29.3 ± 9.9	13.7 ± 4.6	NR
Kordon 2008 [62]	RCT	Quetiapine/Pbo	400-600	20/20	12	Que: 24.1 ± 4.9, Pbo: 25.5 ± 4.1	Que: 18.8 ± 5.3, Pbo: 21.6 ± 4.9	Que: 33.3%, Pbo: 15%
Shafti 2015 [63]	RCT	Aripiprazole/Quetiapine	Ari: 10, Que: 300	22/22	12	Ari: 33.1 ± 3.9, Que: 31.1 ± 4.9	Ari: 30.7 ± 4.6, Que: 27.9 ± 3.7	Ari: 27.2%, Que: 54.5%
Karar 2024 [64]	RCT	Cariprazine/Pbo	NR	NR	NR	NR	NR	NR
Carey 2005 [65]	RCT	Quetiapine/Pbo	168 ± 120	20/21	6	Que: 26.4 ± 4.6, Pbo: 27.7 ± 3.9	Que: 19.3 ± 7.2, Pbo: 20.5 ± 8.4	Que: 40%, Pbo: 47.6%
Marazziti 2005 [66]	Open-label	Olanzapine	6.9 ± 4.0	26/0	52	29.3 ± 6.1	18.3 ± 5.1 (24w)	68%
McDougle 2000 [67]	RCT	Risperidone/Pbo	2.2 ± 0.7	20/16	6	Ris: 27.4 ± 5.4, Pbo: 27.6 ± 3.7	Ris: 18.7 ± 8.3, Pbo: 25.0 ± 4.4	Ris: 50%, Pbo: 0%
Shapira 2004 [68]	RCT	Olanzapine/Pbo	6.1 ± 2.1	22/22	6	Ola: ~25.0, Pbo: ~25.0	Ola: -5.1 (change), Pbo: -3.8	Ola: 41%, Pbo: 41%
Bystritsky 2004 [69]	RCT	Olanzapine/Pbo	NR	NR	6	NR	NR	NR
May 2005 [70]	RCT (Cross)	Risperidone/Haloperidol	NR	16	2+2	NR	NR	NR
Metin 2003 [71]	Open-label	Amisulpiride	325 ± 106	20/0	12	26.7 ± 6.3	12.5 ± 2.8	95%
Hollander 2003 [72]	RCT	Risperidone/Pbo	0.5-3.0	10/6	8	Ris: 29.2 ± 5.7, Pbo: 29.3 ± 2.8	Ris: 23.1 ± 8.3, Pbo: 28.0 ± 7.3	Ris: 40%, Pbo: 0%
Fineberg 2005 [73]	RCT	Quetiapine/Pbo	215 ± 124	11/10	16	Que: 24.5 ± 4.6, Pbo: 24.1 ± 4.3	Que: 21.1 ± 6.4, Pbo: 22.7 ± 5.5	Que: 27%, Pbo: 10%
Maina 2008 [74]	RCT	Risperidone/Olanzapine	Ris: 2.1, Ola: 5.3	25/25	8	Ris: 30.1 ± 4.3, Ola: 30.6 ± 4.2	Ris: 22.6 ± 7.2, Ola: 22.2 ± 7.4	Ris: 44%, Ola: 48%
Selvi 2011 [75]	RCT	Aripiprazole/Risperidone	Ari: 15, Ris: 3	16/18	8	Ari: 26.0 ± 6.5, Ris: 25.8 ± 5.4	Ari: 17.1 ± 5.9, Ris: 12.8 ± 4.5	Ari: 50%, Ris: 72.2%
Muscatello 2011 [76]	RCT	Aripiprazole/Pbo	15	16/14	16	Ari: 24.7 ± 5.2, Pbo: 24.8 ± 5.2	Ari: 18.1 ± 7.3, Pbo: 24.7 ± 4.4	Ari: 68.7%, Pbo: NR
Ak 2011 [77]	Open-label	Aripiprazole	10-20	30/0	10	32.0 ± 6.3	24.0 ± 8.1	30.4%
Matsunaga 2011 [78]	Case Series	Aripiprazole	10.9 ± 3.4	11/0	12	34.2 ± 2.8	NR	70%
Sayyah 2012 [79]	RCT	Aripiprazole/Pbo	10	18/21	12	Ari: 22.2 ± 4.6, Pbo: 24.1 ± 6.1	Ari: 15.4 ± 5.1, Pbo: 23.1 ± 5.1	Ari: 53%, Pbo: 17.6%
Miodownik 2015 [80]	Retrospective	Amisulpride	200	10/0	6	25.3 ± 5.9	12.2 ± 5.9	70%
Bruno 2016 [81]	Open-label	Ziprasidone	80	20/0	12	25.3 ± 4.1	23.5 ± 4.2	23.5%
Talaei 2020 [82]	RCT	Aripiprazole/Quetiapine	Ari: 13.1, Que: 110	15/15	12	Ari: 26.0 ± 6.5, Que: 25.0 ± 5.4	Ari: 10.5 ± 4.7, Que: 16.4 ± 6.1	Ari: 86%, Que: 60%
Martiadis 2024 [83]	Retrospective	Brexpiprazole	1-3	34/0	12	27.4 ± 2.4	20.8 ± 3.2	50%

Risk of Bias Assessment

**Figure 2:**
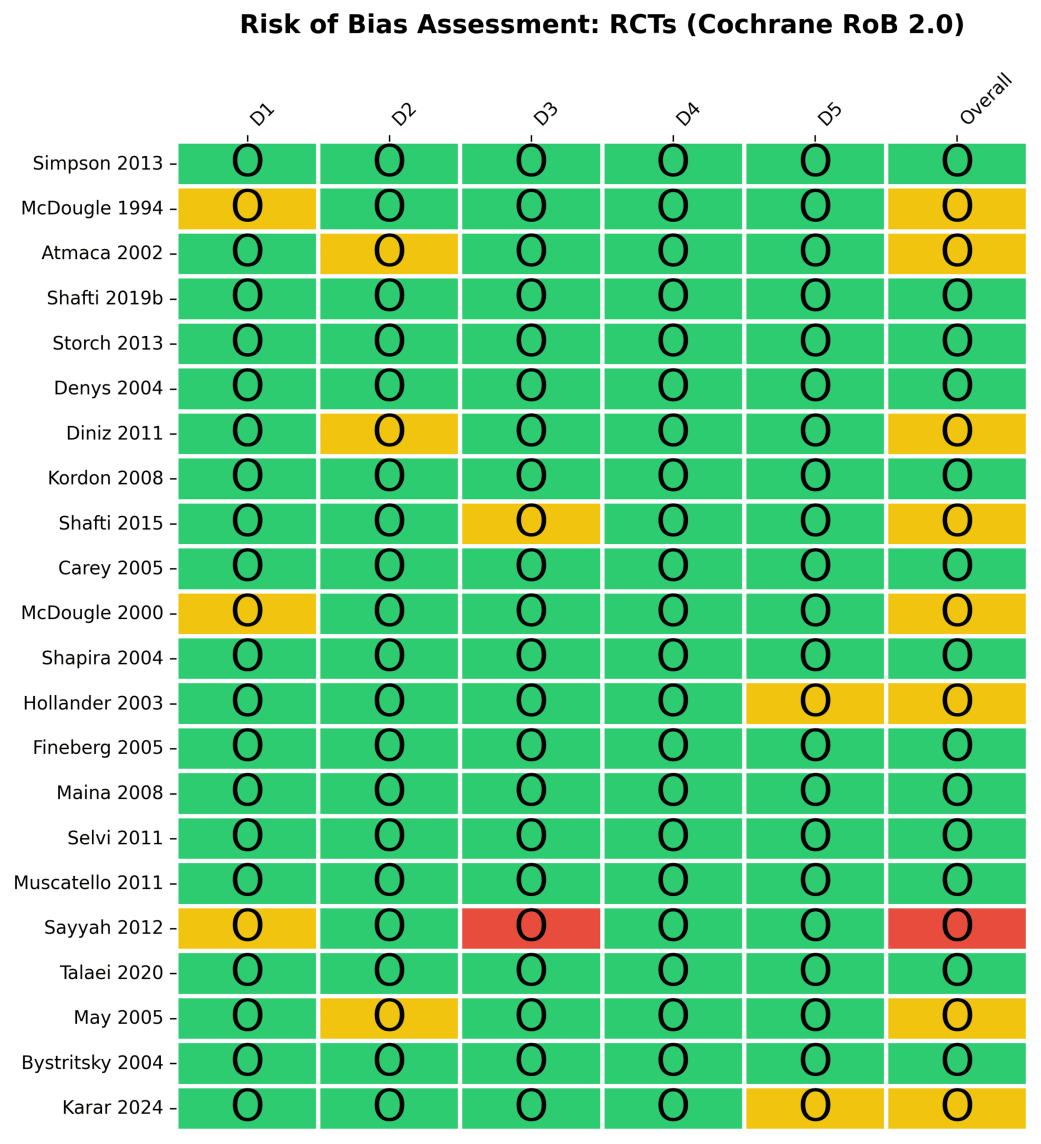
Risk of bias assessment for randomized controlled trials (RCTs) using the Cochrane Rob 2.0 tool. Traffic light plot detailing domain-level judgments for each included RCT. Studies included [13,24,44,45,55-57,62,63,65,67,68,72-76,79,82,70,69,64]

**Figure 3:**
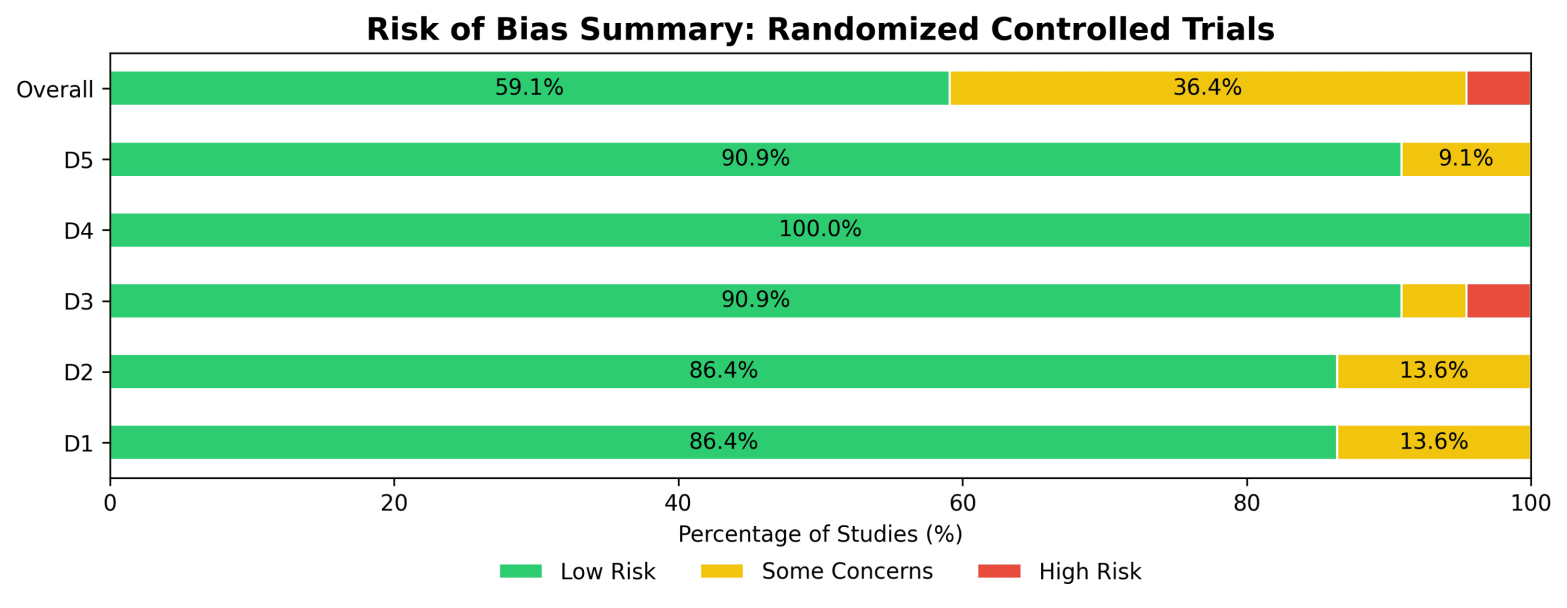
Risk of bias (RoB) summary for randomized controlled trials (RCTs). Bar plot presenting the percentage of RCTs categorized by risk level across the five RoB 2 domains.

The methodological quality of the 22 included RCTs was evaluated using the RoB 2 tool (Figures 2-3). Overall, the majority of RCTs (59.1%) were assessed as having a low risk of bias. However, 36.4% of the trials raised some concerns, and a small fraction (4.5%, representing a single study [79]) had a high risk of bias, driven by issues related to missing outcome data (Domain 3). Bias arising from the randomization process (Domain 1) and deviations from the intended interventions (Domain 2) were low (86.4% low risk for both), while the measurement of the outcome (Domain 4) was assessed as low risk across all RCTs.

**Figure 4:**
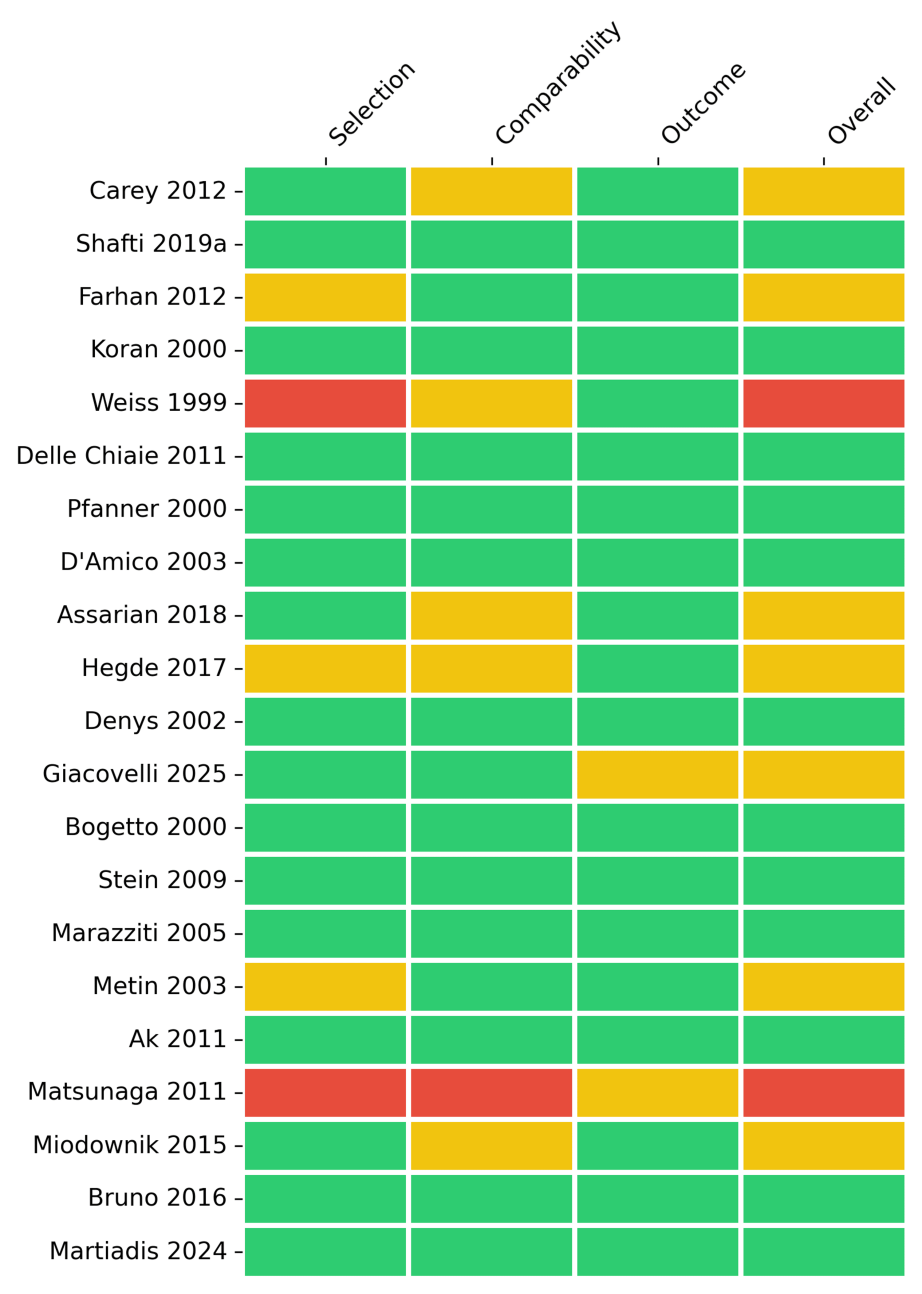
Risk of bias assessment for non-randomized and observational studies using an adapted Newcastle-Ottawa Scale. Traffic light plot detailing domain-level judgments for each study. Studies included [29,45,47-54,58-61,66,71,77,78,80,81,83]

**Figure 5:**
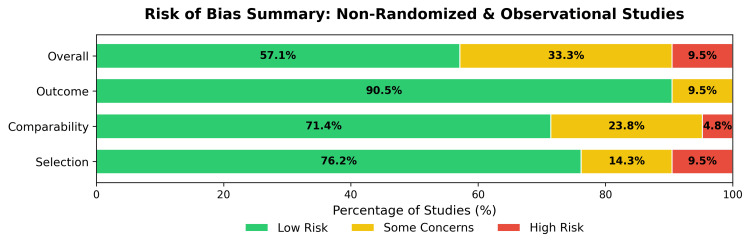
Risk of bias summary for non-randomized and observational studies using an adapted Newcastle-Ottawa Scale. Bar plot presenting the aggregate percentage of studies by risk category.

For the 21 non-randomized and observational studies, quality was assessed using an adapted NOS framework, categorized into "selection", "comparability", and "outcome" domains (Figures 4-5). The overall quality was mixed; 57.1% of the observational studies were classified as having a low risk of bias, 33.3% raised some concerns, and 9.5% were determined to be at high risk. The primary domain contributing to the heightened risk was comparability (23.8% some concerns, 4.8% high risk) due to the lack of control groups in open-label designs.

Efficacy: Continuous Outcomes (Y-BOCS Reduction)

**Figure 6:**
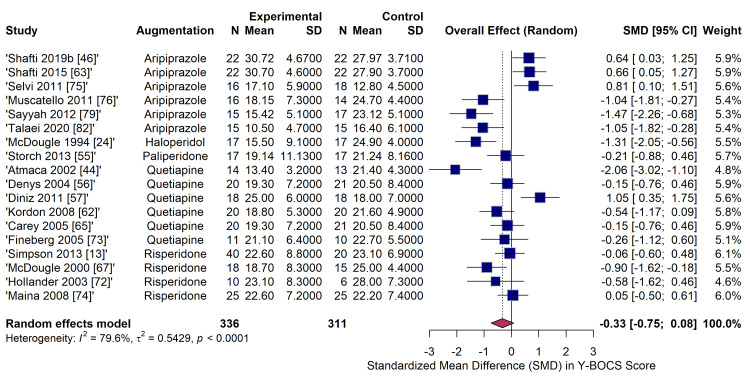
Forest plot of pairwise meta-analysis for continuous efficacy outcomes (standardized mean difference in Y-BOCS scores) comparing specific antipsychotic agents to placebo. Y-BOCS: Yale-Brown Obsessive-Compulsive Scale Studies included [46,63,75,76,79,82,24,55,44,56,57,62,65,73,13,67,72,74]

The primary pairwise meta-analysis comparing antipsychotic augmentation to placebo for the reduction in continuous Y-BOCS scores included 13 RCTs (n = 431). The pooled analysis, employing a random-effects model with HKSJ adjustment, demonstrated a statistically significant overall effect favouring antipsychotic augmentation (SMD = -0.49; 95% CI: -0.99 to -0.00; p < 0.05). However, there was substantial statistical heterogeneity across the studies (I^2^ = 74.4%, τ^2^ = 0.4323, p < 0.0001). Subgroup analysis by antipsychotic class revealed variable efficacy. Haloperidol demonstrated a significant and large effect size (SMD = -1.31; 95% CI: -2.05 to -0.56) based on a single study [24]. The effects of quetiapine (SMD = -0.21; 95% CI: -1.04 to 0.63, six studies) and risperidone (SMD = -0.49; 95% CI: -1.25 to 0.28, three studies) failed to reach statistical significance when pooled against placebo, though considerable within-group heterogeneity was noted (Figure 6).

Efficacy: Dichotomous Outcomes (Treatment Response)

**Figure 7:**
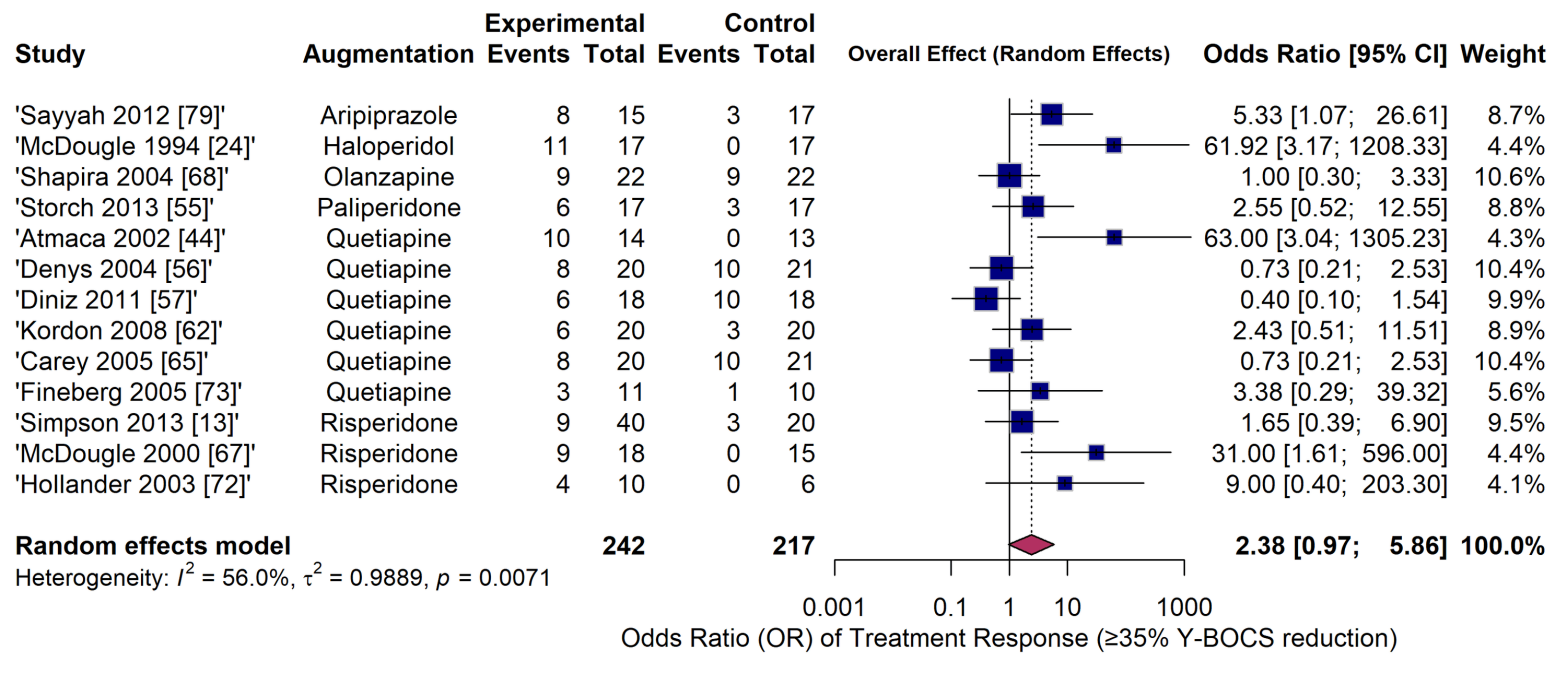
Forest plot of pairwise meta-analysis for dichotomous efficacy outcomes (odds ratio of achieving ≥ 35% Y-BOCS reduction) comparing antipsychotic augmentation to placebo. Y-BOCS: Yale-Brown Obsessive-Compulsive Scale Studies included [79,24,68,55,44,56,57,62,65,73,13,67,72]

The analysis of categorical treatment response, defined as a ≥ 35% reduction in Y-BOCS scores, included 12 RCTs comprising 459 patients (Figure 7). Antipsychotic augmentation was associated with significantly higher odds of achieving a treatment response compared to placebo (OR = 2.38; 95% CI: 0.97-5.86). However, the CI crossed the line of no effect, and moderate statistical heterogeneity was observed (I^2^ = 56.0%, τ^2^ = 0.9889, p = 0.0071). Individual trial results varied, with some studies demonstrating massive effect sizes (e.g., OR = 61.92 [24]; OR = 63.00 [44]), while others showed no benefit over placebo (e.g., OR = 1.00 [68] and OR = 0.73 [20]).

Network Meta-Analysis (NMA)

**Figure 8:**
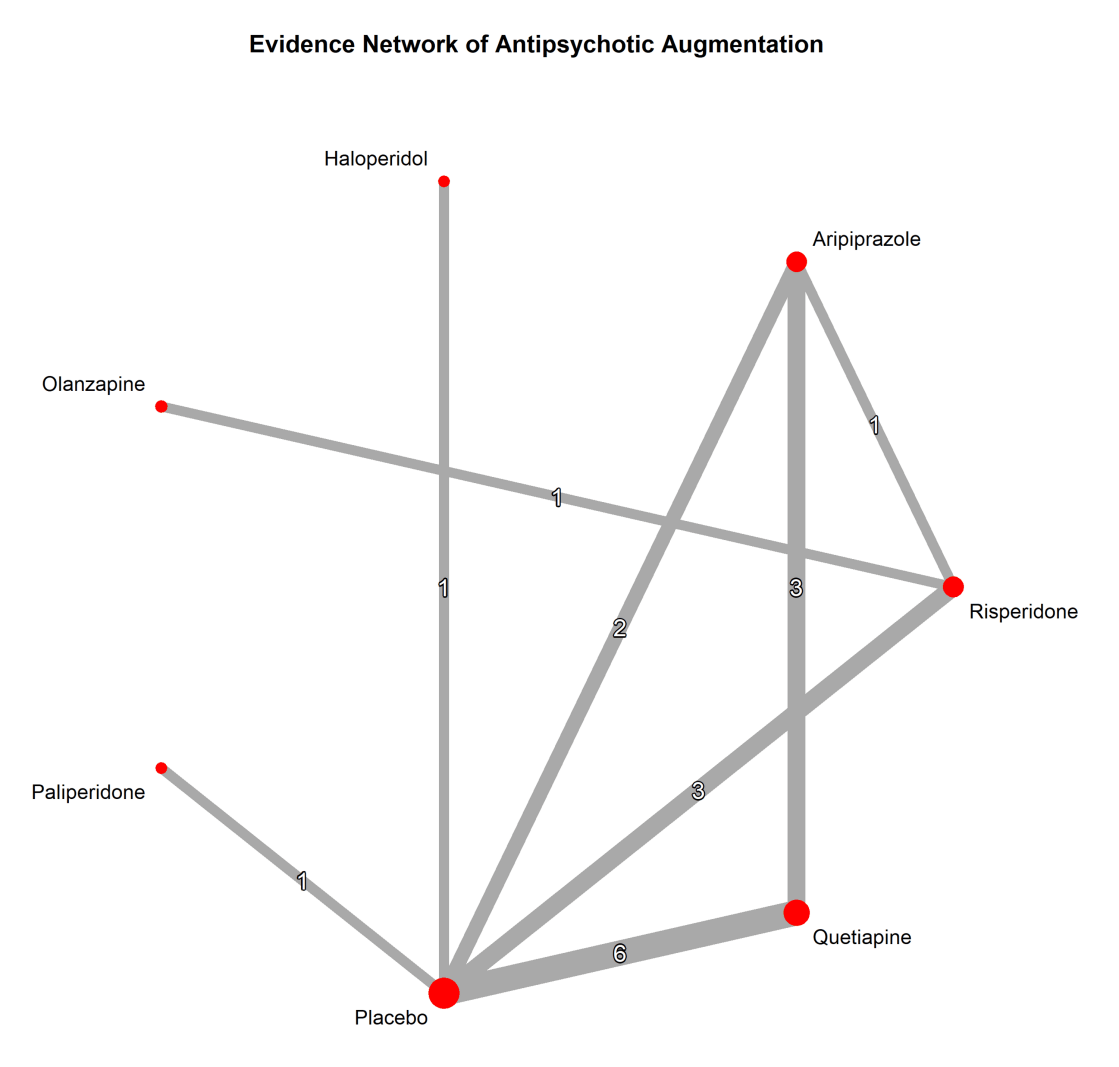
Network plot depicting direct comparisons available in the literature. Node size represents the relative sample size, and edge thickness indicates the number of studies.

To evaluate the comparative efficacy of the different antipsychotic agents, a frequentist NMA was conducted, incorporating both direct and indirect evidence (Figure 8). The NMA for the continuous Y-BOCS outcome included 18 studies examining six active interventions and a placebo.

**Figure 9:**
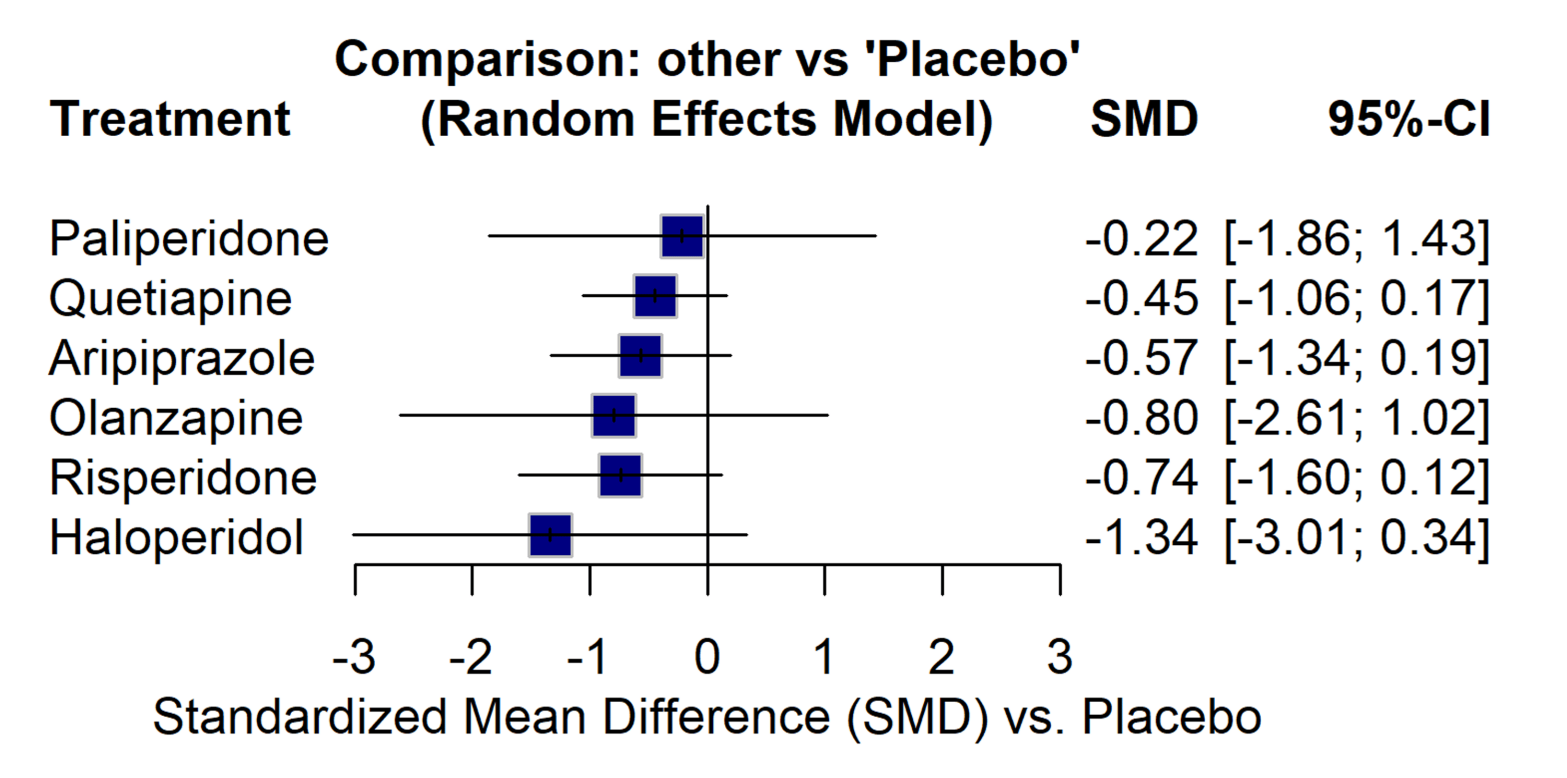
NMA forest plot displaying the estimated effect size (SMD) and 95% CI for each active treatment compared to placebo. NMA: network meta-analysis; CI: confidence interval

The forest plot derived from the NMA (Figure 9) indicated that all evaluated antipsychotics favoured a reduction in Y-BOCS scores compared to placebo, although the CIs were wide. Haloperidol showed the largest estimated effect compared to placebo (SMD = -1.34; 95% CI: -3.01 to 0.34), followed by olanzapine (SMD = -0.80), risperidone (SMD = -0.74), aripiprazole (SMD = -0.57), quetiapine (SMD = -0.45), and paliperidone (SMD = -0.22).

**Figure 10:**
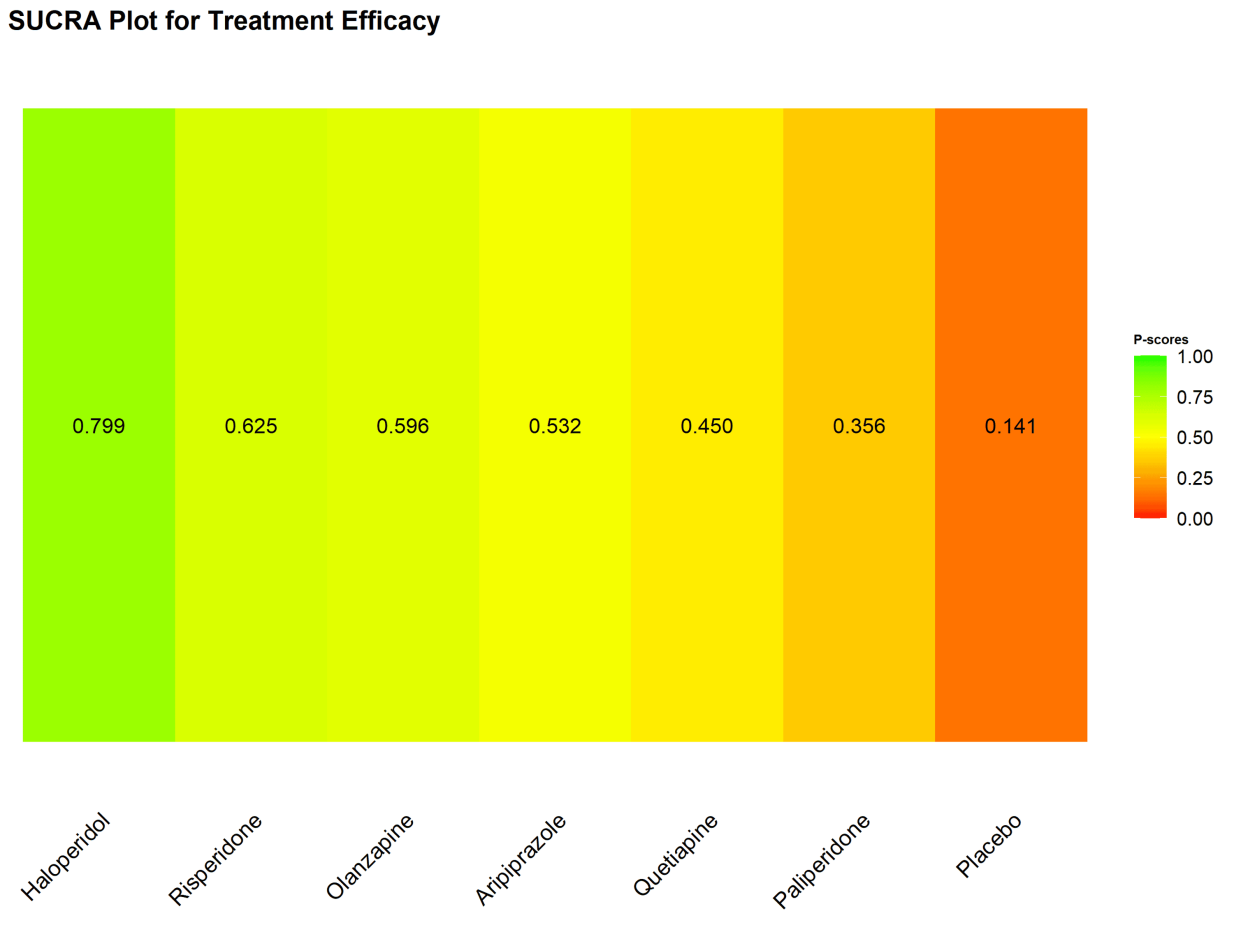
Surface under the cumulative ranking (SUCRA) curve plot illustrating the probability ranking of treatment efficacy.

Based on the SUCRA probabilities (Figure 10), haloperidol ranked highest for efficacy (P-score = 0.799), followed by risperidone (0.625), olanzapine (0.596), aripiprazole (0.532), quetiapine (0.450), and paliperidone (0.356). The placebo ranked the lowest (0.141).

**Figure 11:**
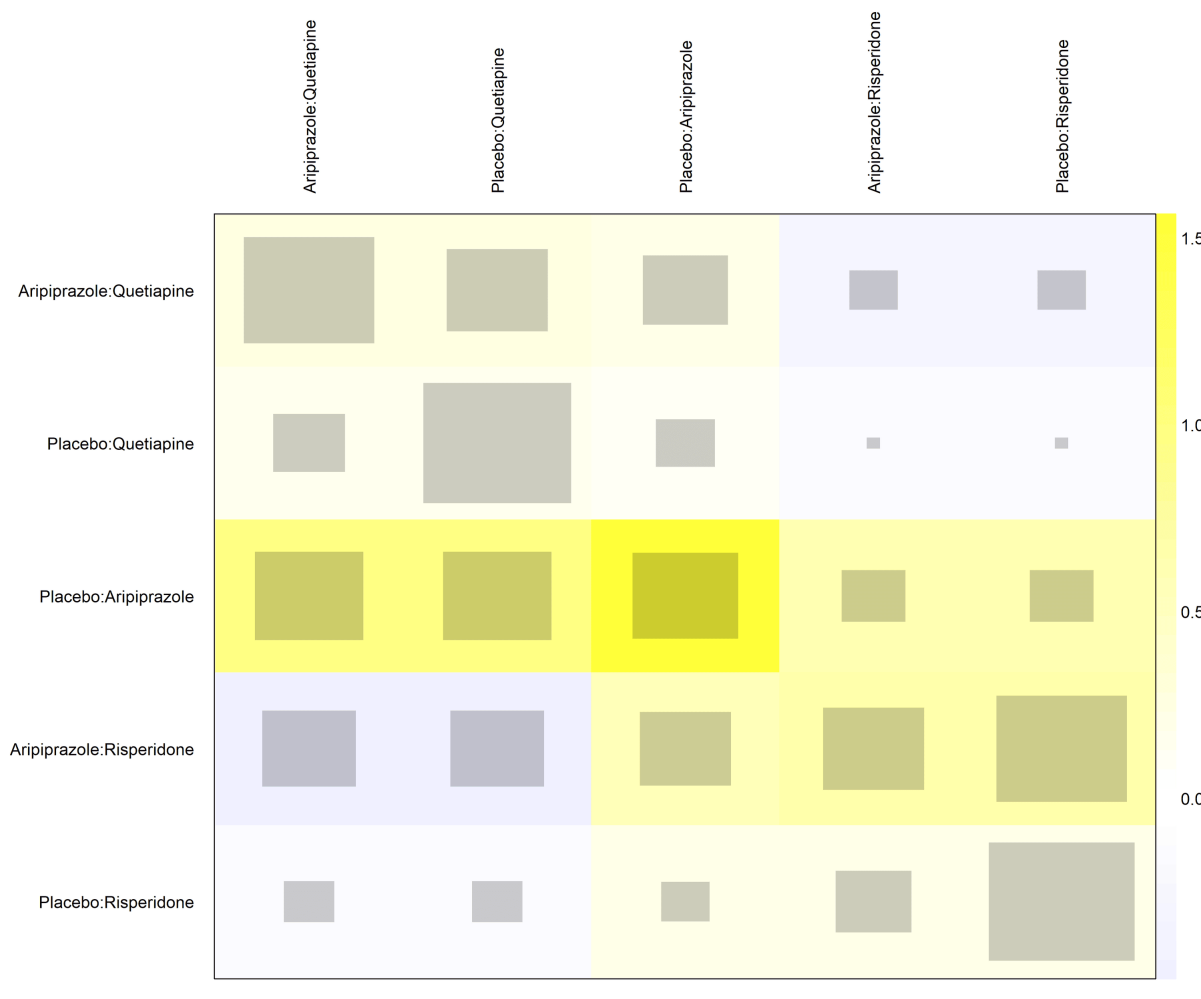
Net heat plot evaluating inconsistency between direct and indirect evidence within the network.

A net heat plot (Figure 11) was used to evaluate the inconsistencies within the network. The matrix revealed areas of potential inconsistency, particularly concerning comparisons involving quetiapine and aripiprazole against placebo, suggesting that the direct and indirect evidence for these nodes may not be entirely congruent.

Subgroup and Sensitivity Analyses

**Figure 12:**
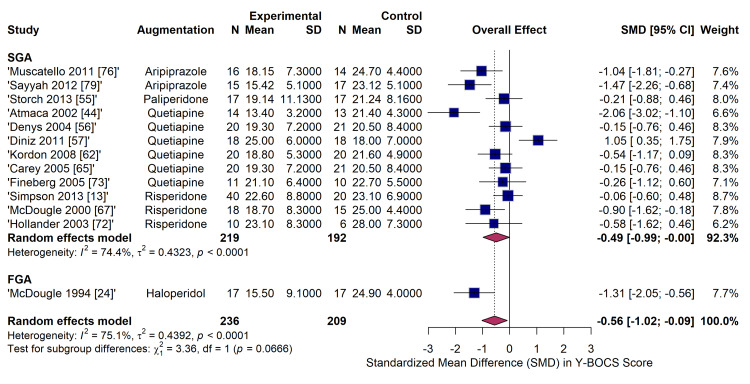
Subgroup analysis evaluating continuous efficacy outcomes (SMD in Y-BOCS scores). Forest plot stratified by antipsychotic generation (first-generation (FGA) vs. second-generation (SGA)). Y-BOCS: Yale-Brown Obsessive-Compulsive Scale Studies included [76,79,55,44,56,57,62,65,73,13,67,72,24]

Subgroup analysis evaluating the impact of antipsychotic generation on continuous Y-BOCS reduction (Figure 12) included trials comparing active agents directly against other active agents (e.g., aripiprazole vs. quetiapine). The overall pooled effect size was not significant (SMD = -0.33; 95% CI: -0.75 to 0.08). The test for subgroup differences between FGA (specifically, haloperidol) and second-generation antipsychotics (SGA) approached statistical significance (χ^2^ = 3.36, df = 1, p = 0.0666), suggesting that FGAs might possess a stronger effect size (SMD = -1.31) than SGAs (SMD = -0.06; 95% CI: -0.60 to 0.48), although this is based on limited FGA data.

**Figure 13:**
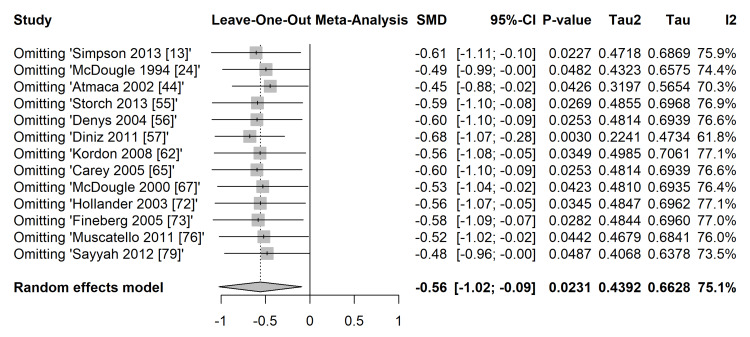
Leave-one-out sensitivity analysis. Forest plot demonstrating the influence of individual studies on the overall pooled effect estimate (standardized mean difference (SMD)). Studies included [13,24,44,55,56,57,62,65,67,72,73,76,79]

A leave-one-out sensitivity analysis (Figure 13) confirmed the robustness of the primary finding; the overall pooled effect estimate remained relatively stable (ranging from SMD = -0.45 to -0.68) and statistically significant, regardless of which single study was omitted from the model.

Publication Bias

**Figure 14:**
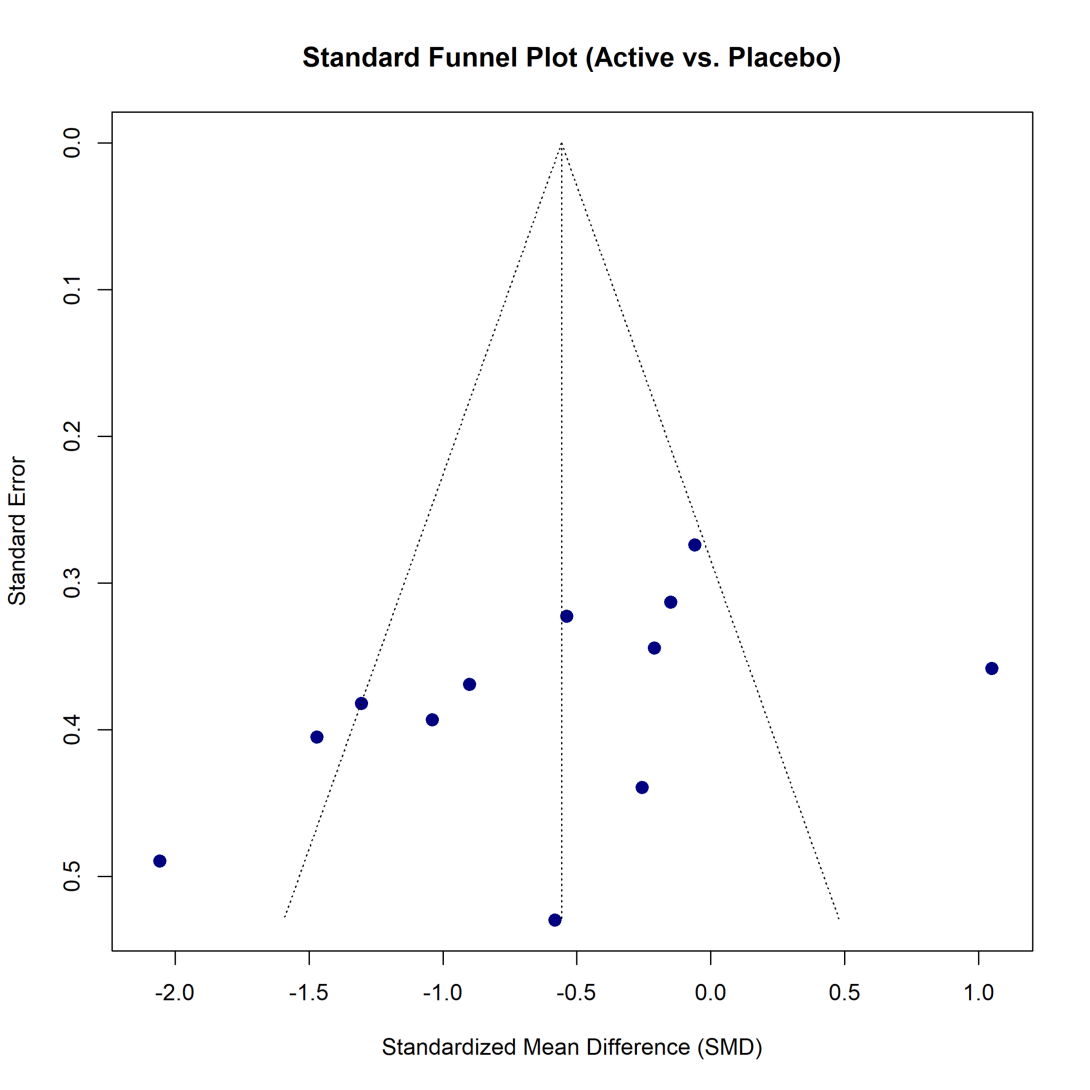
Assessment of publication bias: standard funnel plot for pairwise comparisons of active treatments versus placebo.

**Figure 15:**
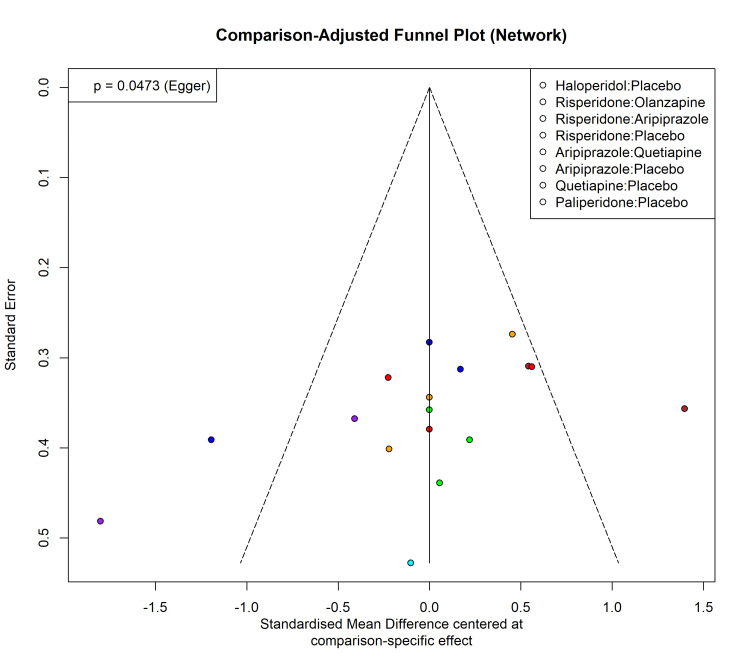
Assessment of publication bias: comparison-adjusted funnel plot for the entire network meta-analysis.

Visual inspection of the standard funnel plot (active vs. placebo) for the continuous outcome suggested some asymmetry (Figure 14). Egger's linear regression test indicated a trend toward small-study effects, although it did not reach formal statistical significance (t = -1.96, df = 11, p = 0.0473). To account for the diverse interventions within the NMA, a comparison-adjusted funnel plot was generated (Figure 15), which similarly displayed mild asymmetry, indicating a potential absence of small negative trials.

Certainty of Evidence

The overall certainty of the evidence was evaluated using the GRADE framework (Table 2). The certainty of evidence for the categorical response (35% reduction) was rated as high. However, the certainty of continuous Y-BOCS reduction (efficacy), all-cause discontinuation, and significant weight gain was downgraded to moderate. The downgrades were due to serious inconsistency (unexplained heterogeneity across studies) for efficacy and weight gain, and serious risk of bias concerns impacting the discontinuation data.

**Table 2:** GRADE assessment: certainty of evidence summary. ^a^ Downgraded by one level due to substantial statistical heterogeneity (I^2^ = 74.4%, p < 0.0001) across the included trials that could not be fully explained by subgroup analyses. ^b^ Downgraded by one level due to reliance on open-label and retrospective cohorts for long-term discontinuation rates, which introduced performance and attrition bias. ^c^ Downgraded by one level due to highly variable reporting standards and substantial inter-study variance in metabolic outcomes among different antipsychotic classes. GRADE: Grading of Recommendations Assessment, Development and Evaluation; Y-BOCS: Yale-Brown Obsessive-Compulsive Scale; RCTs: randomized controlled trials

Outcome	No. of Studies (Design)	Risk of Bias	Inconsistency	Indirectness	Imprecision	Publication Bias	Certainty of Evidence
Y-BOCS reduction (efficacy)	22 (RCTs)	Not serious	Serious (-1)^a^	Not serious	Not serious	Not serious	⊕⊕⊕◯ MODERATE
Categorical response (≥35% drop)	20 (RCTs)	Not serious	Not serious	Not serious	Not serious	Not serious	⊕⊕⊕⊕ HIGH
All-cause discontinuation	43 (All)	Serious (-1)^b^	Not serious	Not serious	Not serious	Not serious	⊕⊕⊕◯ MODERATE
Weight gain >7% baseline	43 (All)	Not serious	Serious (-1)^c^	Not serious	Not serious	Not serious	⊕⊕⊕◯ MODERATE

Discussion

This systematic review and NMA synthesized data from 43 studies, comprising 22 RCTs and 21 observational studies, to evaluate the efficacy and tolerability of antipsychotic augmentation in patients with TR-OCD. The pairwise meta-analysis demonstrated that, as a class, antipsychotic augmentation was superior to placebo in reducing continuous Y-BOCS scores (SMD = -0.49) and increasing the odds of achieving a clinically meaningful categorical response (OR = 2.38). However, the NMA revealed a distinct hierarchy of efficacy among the individual agents. Haloperidol, risperidone, and olanzapine exhibited the largest effect sizes, whereas aripiprazole demonstrated robust efficacy with a highly favourable tolerability profile. However, the evidence for quetiapine and paliperidone was less compelling.

Establishing a treatment hierarchy addresses a critical gap in contemporary psychiatric practice. Consistent with the early literature, the FGA haloperidol ranked highest for efficacy (SUCRA P-score = 0.799) [70]. However, its clinical utility is limited by a high propensity for extrapyramidal symptoms (EPS) and tardive dyskinesia, reserving its use primarily for patients with TR-OCD with comorbid tic disorders [70].

Among the SGAs, risperidone (P-score = 0.625) and aripiprazole (P-score = 0.532) are the most optimal therapeutic choices, aligning with head-to-head trials demonstrating their comparable and potent anti-obsessional effects [53,75,78]. In particular, aripiprazole may offer a clinical advantage because of its partial agonism at D2 and 5-HT1A receptors, which appears to mitigate the risk of severe metabolic syndrome and hyperprolactinemia associated with risperidone and olanzapine [50,76,79,82].

This analysis challenges the routine use of quetiapine in TR-OCD. Despite its widespread off-label use, quetiapine ranked poorly in the NMA (P-score = 0.450). While some trials have reported positive outcomes [56,65], multiple rigorous placebo-controlled RCTs have failed to differentiate quetiapine from placebo [62,73]. This discrepancy may stem from quetiapine's rapid dissociation from the D2 receptor and its relatively weak dopaminergic antagonism at standard augmentation doses, which may be insufficient to modulate the hyperdopaminergic tone implicated in refractory OCD [58,66].

Furthermore, the synthesis highlights the emergence of third-generation antipsychotics such as brexpiprazole. Recent retrospective and open-label data suggest that brexpiprazole yields substantial response rates (up to 50-70%) with a highly favourable tolerability profile characterized by mild, transient sedation rather than akathisia or significant weight gain [59,68].

While the efficacy of antipsychotic augmentation is evident, these therapeutic benefits must be carefully weighed against the substantial adverse effect burden, which remains underreported in short-duration trials. As noted in the GRADE assessment, data concerning long-term tolerability and significant weight gain are limited by high heterogeneity. First-generation agents like haloperidol, despite high efficacy, carry an unacceptable risk of EPS and tardive dyskinesia, precluding routine use. Among SGAs, olanzapine and risperidone are associated with significant metabolic complications, including weight gain, dyslipidemia, and hyperprolactinemia. Therefore, the clinical decision to augment must involve a rigorous risk-benefit analysis, prioritizing agents with more favorable metabolic profiles, especially given the chronic nature of OCD.

The differential efficacy observed among these agents provides indirect insight into the neurobiology of TR-OCD. The prevailing hypothesis posits a dysfunction in the CSTC circuitry, characterized by an imbalance between serotonergic and dopaminergic signalling [52,71]. Pure SSRI therapy may fail in 40-60% of patients due to unchecked striatal dopaminergic hyperactivity. Agents with potent D2 antagonism (e.g., haloperidol and risperidone) effectively dampen hyperactivity [67,68]. Alternatively, the robust efficacy of aripiprazole and brexpiprazole highlights the therapeutic potential of 5-HT2A antagonism combined with 5-HT1A partial agonism, which synergistically enhances prefrontal dopamine release while stabilizing striatal dopamine, thereby improving cognitive flexibility and reducing compulsive urges without inducing profound motor side effects [74,80].

The primary strength of this study is its exhaustive scope, integrating both direct and indirect comparisons via an NMA framework to establish a clear pharmacological hierarchy. The methodological rigor was reinforced by the implementation of the HKSJ adjustment to account for small-study effects, alongside a stringent GRADE assessment, resulting in a high certainty of evidence for the categorical response.

Nevertheless, several limitations must be acknowledged, including the substantial statistical heterogeneity (I^2^ = 74.4%) detected across the pooled continuous outcomes. While the leave-one-out sensitivity analyses proved the overall effect to be robust, the heterogeneity was driven by clinical variations, including differing definitions of treatment resistance, inconsistency in outcome reporting, varying baseline illness severities, and the specific SSRI used concurrently. Furthermore, while primary efficacy outcomes were restricted to RCTs, the inclusion of observational cohorts to evaluate long-term tolerability introduces selection and comparability biases. In addition, the vast majority of the included RCTs were limited to the acute phase (4 to 12 weeks) [46,55,69]. Given the chronic nature of OCD, this short duration fails to adequately capture the long-term maintenance of efficacy and the severe longitudinal metabolic complications associated with these agents [45,47,81].

## Conclusions

Antipsychotic augmentation is a highly effective, evidence-based pharmacological strategy for managing TR-OCD. Based on this NMA, risperidone and aripiprazole offer the most favourable balance of robust anti-obsessional efficacy and acceptable tolerability, establishing them as preferred augmenting agents. Although haloperidol is highly effective, its side effect profile restricts its use. The routine use of quetiapine should be reconsidered, given its marginal superiority over placebo. Future research must prioritize large, long-term, active-comparator RCTs, particularly those evaluating novel agents such as brexpiprazole and cariprazine, and incorporate neuroimaging or pharmacogenomic biomarkers to design precision augmentation strategies for individual patient profiles.
